# 

**Published:** 1898-11

**Authors:** 


### PERSONALS.

Dr. David S. White, of Columbus, the newly appointed member of the Ohio State Board of Veterinary Medical Examiners, has been elected Secretary of the board in that State. Dr. White fills the position of Dean of the Ohio State University, Veterinary Department.

Dr. S. G. Hendren, of York, Pa., is now visiting his family at Roxborough, Philadelphia. Dr. Hendren was temporarily attached to one of the engineer corps of the army.

Drs. Felber and Œsterling, graduates of the Veterinary Department of the University of Pennsylvania, class of 1895, were visitors to Philadelphia during the Peace Jubilee in October.

Dr. B. F. Minnick, of Columbia, Pa., was a visitor to the City of Brotherly Love during the Peace Jubilee in October.

Dr. J. C. McNeil, of Pittsburg, Pa., was a visitor to Philadelphia's Peace Jubilee in October, coming as an official of the "Smoky City." He was a guest of the city officials of Philadelphia.

Dr. W. H. Pethick holds the position of Dominion Government Veterinary Inspector for Prince Edward Island. He very strongly approves the change to the American Veterinary Medical Association, and has already become an applicant for association honors.

Dr. W. H. Wheeler, of Stamford, N. Y., finds his location a congenial one and is rapidly gaining a clientage in that field.

Dr. A. Mitchell, of Skaneateles Falls, N. Y., has just been mustered out from the battery of his district, having enlisted at Port Chester, N. Y., at the outbreak of the Hispano-American War.

Dr. H. W. Stedman, of Springfield, Mass., has recently located at Barton, Vermont.

Dr. E. S. Walmer, of Washington, D. C., and Dr. E. H. Landes, of Camden, N. J., were sent to the army posts in Florida early in October, to eliminate glanders and farcy from among the horses stationed there.

Dr. L. A. Reefer, of Wheeling, W. Va., was solicited by our Government to accept a position at Tampa and Porto Rico, but declined the offers sent.

Dr. Louis O. Lusson, of Ardmore, officiated as marshal of the ninth division of the Civic Day parade at the Peace Jubilee of Philadelphia, in October.

Dr. Albert E. Cunningham, of the class of 1898, Veterinary Department of the University of Pennsylvania, has opened a veterinary hospital at 215 Huntington Street, Cleveland, Ohio. Dr. Cunningham passed the Pennsylvania State Board of Veterinary Medical Examiners in June last, and received their license, and has now been granted a license by the Ohio State Board.

Dr. N. B. Rhodes, of Tampa, Fla., treated many of the officers' horses in his private hospital, because of lack of facilities being provided for by those charged with these duties. It was easier to ignore competent veterinary advice and drive it from the army service, lest some stubborn and conceited minor officer's power should be questioned or disciplined.

Dr. Leonard Pearson addressed the students' society at the Veterinary Department of the University of Pennsylvania, October 21, 1898, on the subject of "Conformation of the Horse."

Dr. M. J. Treacy, of the Eighth Cavalry, formerly of Ft. Meade, S. Dak., has been stationed at Huntsville, Ala., on waiting orders for probable transport to Cuba or Porto Rico.

Dr. J. C. Saile, of Passaic, has changed his location to Bloomfield, N. J.

Dr. A. J. Savage, of Colorado Springs, Col., by dint of becoming with many others a determined fire-fighter for many hours, succeeded in saving his plant from the great conflagration there in October.

Among those taking the Civil Service Examinations for the Bureau of Animal Industry at Philadelphia in October were to be noted Drs. Jas. Beatty, B. M. Underhill, and J. H. McNeall.

Prof. Leonard Pearson, of the Veterinary Department of the University of Pennsylvania, addressed the Farmers' Institute at Emporium, Pa., in October, on "Dairying in Denmark."

Secretary J. W. Petty, of the North Carolina Veterinary Medical Association, is just recovering from a long and serious illness.

Dr. J. C. Meyer, Jr., of Cincinnati, Ohio, was among the Sir Knights Templar at their triennial conclave at Pittsburg in October, and included a visit to Washington, D. C., Philadelphia, and Reading, Pa., before his return home.

Dr. Jas. W. Sallade, of Pottsville, Pa., has taken out letters patent upon a commercial product called "laundrine," and established a depot for its production and sale.

Dr. Edwin J. Castle, of Methuen, Mass., conducts a drug-store in connection with his practice.

Dr. S. S. Whitbeck, Decorah, Iowa, spends a portion of his time lecturing to the farmers' institutes throughout the State.

Dr. T. A. Bown, of Chariton, Iowa, has become largely interested in the shipment of horses to the Eastern markets.

Dr. J. A. Mitchell, one of the Indiana delegates and a close candidate for Central Vice-President, will lecture to the horseshoers of Evansville this winter.

Dr. Jos. Johnson, of the class of '98, Veterinary Department, University of Pennsylvania, recently located at West Chester, has succeeded to the practice of Dr. Hogg, of Kirkwood.

Dr. J. McBirney still continues at the head of the special bureau work in Page County, Iowa, investigating and directing the control of hog-cholera.

Dr. J. Greer, formerly of Colorado Springs, is now assistant to Dr. S. Bock, of Denver, Colorado.

Dr. F. W. Hunt, a graduate of the class of '88 of the American Veterinary College, is now located in Denver, Colorado.

Veterinarian James Murray, of Detroit, Michigan, enjoys the possession, also, of a degree in human medicine.

Dr. Richard W. Hewitt, of Bridgeton, N. J., has retired from the practice of veterinary science and turned his attention to commercial pursuits.

Dr. E. S. Muir, of Philadelphia, in connection with his practice, conducts the Kensington Veterinary Hospital.

Dr. T. W. Scott, formerly of Nashville, recently located at Clarksville, Tenn., where, in conjunction with a veterinary infirmary, he conducts a shoeing-forge.

Dr. G. Howard Davidson, of Millbrook, N. Y., has been reelected Secretary of the National Association of Exhibitors of Livestock.

Dr. D. Ryder, formerly of Chambersburg, Pa., is now located at Tampa, Fla., under special employment of the Government in connection with the horses in the army department.

